# Genome-Wide Reassortment Analysis of Influenza A H7N9 Viruses Circulating in China during 2013–2019

**DOI:** 10.3390/v14061256

**Published:** 2022-06-09

**Authors:** Dongchang He, Xiyue Wang, Huiguang Wu, Xiaoquan Wang, Yayao Yan, Yang Li, Tiansong Zhan, Xiaoli Hao, Jiao Hu, Shunlin Hu, Xiaowen Liu, Chan Ding, Shuo Su, Min Gu, Xiufan Liu

**Affiliations:** 1 Animal Infectious Disease Laboratory, College of Veterinary Medicine, Yangzhou University, Yangzhou 225009, China; virolog@outlook.com (D.H.); bioyusy@outlook.com (X.W.); hgwu80@163.com (H.W.); wxq@yzu.edu.cn (X.W.); bioyyy@outlook.com (Y.Y.); liyang1631@163.com (Y.L.); biocreater@outlook.com (T.Z.); xlhao@yzu.edu.cn (X.H.); hujiao@yzu.edu.cn (J.H.); slhu@yzu.edu.cn (S.H.); xwliu@yzu.edu.cn (X.L.); 2 Jiangsu Co-innovation Center for Prevention and Control of Important Animal Infectious Diseases and Zoonosis, Yangzhou University, Yangzhou 225009, China; shoveldeen@shvri.ac.cn; 3 Jiangsu Key Laboratory of Zoonosis, Yangzhou University, Yangzhou 225009, China; 4 Department of Avian Diseases, Shanghai Veterinary Research Institute, Chinese Academy of Agricultural Sciences, Shanghai 200241, China; 5 College of Veterinary Medicine, Nanjing Agricultural University, Nanjing 210095, China; shuosu@njau.edu.cn

**Keywords:** avian influenza virus, H7N9, highly pathogenic, diversity, reassortment, tanglegram

## Abstract

Reassortment with the H9N2 virus gave rise to the zoonotic H7N9 avian influenza virus (AIV), which caused more than five outbreak waves in humans, with high mortality. The frequent exchange of genomic segments between H7N9 and H9N2 has been well-documented. However, the reassortment patterns have not been described and are not yet fully understood. Here, we used phylogenetic analyses to investigate the patterns of intersubtype and intrasubtype/intralineage reassortment across the eight viral segments. The H7N9 virus and its progeny frequently exchanged internal genes with the H9N2 virus but rarely with the other AIV subtypes. Before beginning the intrasubtype/intralineage reassortment analyses, five Yangtze River Delta (YRD A-E) and two Pearl River Delta (PRD A-B) clusters were divided according to the HA gene phylogeny. The seven reset segment genes were also nomenclatured consistently. As revealed by the tanglegram results, high intralineage reassortment rates were determined in waves 2–3 and 5. Additionally, the clusters of PB2 c05 and M c02 were the most dominant in wave 5, which could have contributed to the onset of the largest H7N9 outbreak in 2016–2017. Meanwhile, a portion of the YRD-C cluster (HP H7N9) inherited their PB2, PA, and M segments from the co-circulating YRD-E (LP H7N9) cluster during wave 5. Untanglegram results revealed that the reassortment rate between HA and NA was lower than HA with any of the other six segments. A multidimensional scaling plot revealed a robust genetic linkage between the PB2 and PA genes, indicating that they may share a co-evolutionary history. Furthermore, we observed relatively more robust positive selection pressure on HA, NA, M2, and NS1 proteins. Our findings demonstrate that frequent reassortment, particular reassorted patterns, and adaptive mutations shaped the H7N9 viral genetic diversity and evolution. Increased surveillance is required immediately to better understand the current state of the HP H7N9 AIV.

## 1. Introduction

Influenza A virus carries enveloped genomes consisting of eight gene segments of single-stranded ribonucleic acid molecules. When two viruses co-infect the same cell, the progeny virions may contain heterogeneous segments from different genomic sources. This process is known as reassortment, which is crucial for viral evolution [1] and adaptation [2]. Reassortment creates epidemiologically significant variants that can influence virus propagation, illness severity, antiviral resistance, and vaccine efficacy [3,4,5]. The combination of reassortment and genomic mutations increases the viral diversity while also shaping their short-term evolution [6,7,8]. Particularly, when a reassortant contains novel antigens against the naive population and is able to achieve cross-host transmission from other species to humans (host jump), it meets the critical requirements for a pandemic with high potential. However, most reassortants are less fit than either parent and then cleaned by the purifying selection [9]. Occasionally, the reassortment leads to the formation of a gene combination with mutations under a particular set of selection pressures, resulting in improved fitness [9] and the potential of a pandemic. Almost all influenza viruses responsible for human pandemics are thought to have evolved through reassortment [2,3,5,10,11].

The reassorted avian influenza H7N9 virus emerged in 2013 with antigenic novelty, resulting in substantial human mortality (30–50%) and posing a significant threat to public health [12]. The six internal genes of the H7N9 virus were derived from at least two different H9N2 virus lineages, and the H7 and N9 genes came from wild birds [13,14,15,16,17]. Nonetheless, the H7N9 virus has continually undergone reassortment with other subtypes of AIVs since its emergence, such as seasonal influenza A virus H1N1, H3N2, and even influenza B virus [18,19,20], H5N6 [21,22], H6Ny [22,23], H9N2 [23,24,25,26,27,28], and even H7N9 itself [29]. As a result, multiple novel viruses were generated, including H7N2 [30], H7N3 [31], H7N4 [32], and H7N6 [33]. However, most of these reassortants were transient except for the progeny viruses with the H9N2 (especially the G57 or S genotype H9N2 [34]). The H9N2 virus facilitated the genesis, adaptation, and prevalence of the H7N9 virus [15,35]. Significantly, reassortment with H9N2 has contributed to the onset of the fifth epidemic wave, which is the largest H7N9 outbreak in humans [36]. Due to the ease of notifying the intersubtype reassortment with NA, the actual reassortment events and patterns involving the internal segments may largely remain unknown.

In China, two lineages have been identified and named based on the outbreak sources: the Yangtze River Delta (YRD) and the Pearl River Delta (PRD). The YRD lineage was found to be responsible for the majority of H7N9 outbreaks [17]. Based on this existing nomenclature system, we further divided the H7N9 into eight genotypes. Then, we performed systemic genomic analyses on the eight segments of the H7N9 virus from wave 1 to wave 7. Our study enabled us to estimate the frequency of reassortment and particular patterns among the H7N9 virus. Therefore, we conclude that reassortment and mutation co-shaped the evolution and epidemiology of the H7N9 virus. Monitoring reassortment and mutations in H7N9 virus predominance in chicken flocks is critical for preventing the virus from spreading to humans.

## 2. Materials and Methods

### 2.1. Sequences Collection

We downloaded the H5, H6, and H9 subtypes of avian influenza viruses circulating in Asia from 2010 to 2019 from the online databases of the *Influenza Virus Resource, NCBI Influenza Research Database*, and *Global Initiative on Sharing All Influenza Data* (accessed on 1 September 2019). We downsampled the collected sequences with a 0.99 threshold by CD-HIT (http://weizhong-lab.ucsd.edu/cdhit-web-server/cgi-bin/index.cgi, accessed on 1 October 2019) to minimize the computation resource consumption. Then, sequences were aligned using MAFFT (v7.453) [37] and trimmed to keep the coding region. As a result, polymerase basic protein 2 (PB2) (*n* = 1125), polymerase basic protein 1 (PB1) (*n* = 1118), polymerase acidic protein (PA) (*n* = 1069), matrix protein (M) (*n* = 883), nucleoprotein (NP) (*n* = 1000), and non-structural protein (NS) (*n* = 884) gene sequences were obtained. For the genomic sequences of H7N9, the cd99 and cd999 datasets were adopted from our previous study [38].

### 2.2. Phylogenetic Analyses and Diversity Estimates

We used Bayesian evolutionary analysis sampling trees (BEAST, v1.10.4) [39] program to interpret the (cd99 dataset) phylogenetic tree and evolutionary rate (ucld.mean) of eight segments. The substitution model was determined by ModelFinder in PhyloSuite [40] according to the optimality Bayesian information criterion (BIC). The uncorrelated relaxed molecular clock model was used, and the Bayesian SkyGrid coalescent model was set as the tree prior [41]. Bayesian Markov chain Monte Carlo (MCMC) chains were set to 200 million generations with samples for every 20,000 steps to create the XML files. Each XML file was executed at least three times. Subsequently, the log files were assessed by Tracer (v1.7.1), and the effective sample size (ESS) values greater than 200 were accepted. Lastly, the maximum clade credibility (MCC) trees with median node heights were generated after the first 10% burn-in by TreeAnnotator (v1.10.4). The HA and NA MCC phylogenetic trees were adopted from our previous study [38]. Meanwhile, we performed the diversity test with the H7N9 cd999 dataset by the MEGA X (v10.2) to estimate the nucleotide diversity.

### 2.3. Phylogenetic Clusters Classification

Phylogenetic clusters were firstly classified based on the MCC trees of eight segments. Phylopart (v2.1) is a methodology for large-scale phylogeny partition [42]. We used it to define monophyletic clades with posterior probabilities of ≥90% and a median genetic distance threshold for clusters of 20% [15]. The assignment to these clusters was subsequently manually merged based on cluster tips containing ≤ 3 and failed to cluster. Then, each cluster was assigned a unique number based on the increasing tree topology. The remaining 7 MCC trees were assigned to clusters using the same processes. The H7N9 genotypes were classified using the HA clusters result and a previous investigation [17]. Phylogenetic tree and heatmap were plotted and visualized using ggtree [43,44].

### 2.4. Reassortment Detections

#### 2.4.1. Intersubtype Reassortment

To investigate intersubtype reassortments across the six internal segments (PB2, PB1, PA, NP, M, and NS), we integrated the main subtypes (H5, H6, H7, and H9) of avian influenza viruses that circulated from 2010 to 2019. Multiple sequence alignments were performed with MAFFT [37]. Then, phylogenetic tree construction was performed by Mrbayes (v3.2.7a) [45]. The GTR+G+I model parameters were incorporated into the nexus file. Then, the nexus files were implemented in MrBayes by running 20 million MCMC chains with a sampling frequency of 5000. The phylogenetic trees were visualized and plotted using ggtree and ggtreeExtra [43,44].

#### 2.4.2. Entanglegram and Untanglement

Tanglegram is a visual method to draw an association between two phylogenetic trees with identical tip labels. Tanglegram is also known as the incongruence tree. Theoretically, when the reassortment event is absent, the twines connect the tanglegram in a noncrossing or horizontally way [46]. To uncover the H7N9 AIV intrasubtype reassortment, we used the backronymed adaptable lightweight tree import code (Baltic) to generate incongruence visualization of eight H7N9 AIV MCC trees. The phylogenetic position of each strain was traced and colored according to the HA clusters across all segments. Entanglement was generated from an adapted script (https://github.com/dven42/phylogenetic-incongruence, accessed on 1 June 2021) and modified to visualize the positions of specific isolates for our study.

Untanglegram is a visual method to minimize crossings of hybridization networks between the tanglegram by rotating their branches around ancestral nodes [47]. There is an arrangement of their branches such that the association edges do not intersect if the topologies of the two phylogenies are entirely concordant [47]. The untanglegram phylogeny reveals the extent of reassortment between the HA and seven other segments (PB2, PB1, PA, NA, NP, and M). The identical tips were connected across the trees. Tips and connecting lines were colored according to the HA clusters. Untanglements were plotted with Dendextend using the step1side untangle method [48] by fixing the left tree (HA) and rotating the right tree (the other segments) to generate the minimization hybridization tanglegram in R (v4.0.5). The bigger the untanglement value is, the worse the reassortment between the paired tree.

#### 2.4.3. Quantified Investigation Reassortment Events

We investigated the reassortment events over the entire genome by combined approaches of Graph Incompatibility based Reassortment Finder (GiRaF, v1.02) [49] and Recombination Detection Program (RDP, v5.05) [50]. In brief, Mrbayes assessed multiple trees per segment for incompatible splits using the GiRaF. After twenty million generations and sampling every 5000 steps, a burn-in of 25% samples was established to obtain trees and subsequently used for input files. Reassortment events with a confidence level greater than 0.9 were deemed accurate. Meanwhile, we also investigated the reassortment events using the RDP from the concatenated segments [51]. The algorithms incorporate the RDP, GENECONV, BOOTSCAN, MAXCHI, CHIMAERA, SISCAN, and 3SEQ techniques. At least four of the seven detection methods with a *p*-value of 10^−6^ were acknowledged as recombination events. Only strains confirmed concurrently by GiRaF and RDP were considered putative reassortment events.

BEAST2/CoalRe (v0.05) [51] estimated intralineage reassortment networks between H7 and N9. Three runs of 200 million MCMC sampling steps were performed following the tutorial (https://taming-the-beast.org/tutorials/Reassortment-Tutorial/, accessed on 1 June 2021). The embed segment tree was drawn using icytree (https://icytree.org/, accessed on 1 October 2021) to depict the reassortment network between HA and NA.

### 2.5. Multidimensional Scaling

We used multidimensional scaling (MDS) to analyze each viral segment’s tree-to-tree branch variation and investigate the overall level of cross-correlation between all segments in a two-dimensional space. Generally, the last 500 phylogenetic trees for each segment were obtained from independently sampled trees in BEAST. The statistical tree-to-tree variation in branch lengths was calculated. Then, the MDS statistics analysis was performed in the R (v4.0.5) to determine the cross-correlation of all segments in two-dimensional space. Only the first two scaling dimensions were plotted using the ggplot2 [52] package for visualization. Theoretically, the viral segments that share similar evolutionary histories occupy similar locations in the two-dimensional Euclidean space where the cloud of points should overlap. Clouds of points in the MDS plot indicate phylogenetic uncertainty based on 500 sampled trees. In contrast, segments are expected to exhibit uncorrelated features due to their unlinked evolutionary histories in response to reassortment. Scripts for the MDS calculation were obtained from Doctor Maude Jacquot et al. [4,53] and modified for this study.

### 2.6. Selection Pressure Analysis

We quantified the selection pressures acting on the 10 major protein-coding regions (M and NS, respectively, encode at least two proteins) under different models. Before selection analysis, the best-fit model of nucleotide substitution was obtained from Modelfinder [54] based on Bayesian information criterion (BIC) for each segment. The maximum likelihood (ML) phylogenetic tree was interpreted using IQtree in PhyloSuite (v1.2.2). Subsequently, single-likelihood ancestor counting (SLAC) [55], fast unconstrained Bayesian approximation (FUBAR) [56], fixed-effects likelihood (FEL), and mixed-effects model of evolution (MEME) [57] were used to infer sites under episodic or pervasive natural selection on each coding protein. Finally, we recommend the significance levels for FEL (*p* < 0.1), SLAC (*p* < 0.05), MEME (*p* < 0.05), and FUBAR (posterior probability > 0.9). All methods were implemented in the HyPhy (v2.5.2) [58] on a high-performance computing cluster. The selection results of HA and NA were adopted from our previous study [38].

## 3. Results

### 3.1. The Surface Glycoproteins Have Faster Evolutionary Rates but Less Genetic Diversity

**Figure 1:**
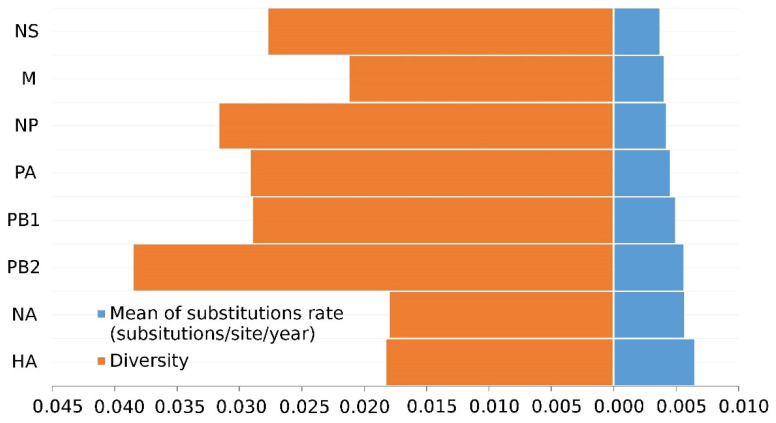
Diversity and evolutionary rates of H7N9 eight gene segments.

Nucleotide analyses revealed that the internal segments have higher diversity than the surface glycoproteins (Figure 1). PB2 (3.86 × 10^−3^) has the highest genetic diversity among all segments, exhibiting a highly heterogeneous genome. The HA gene (1.83 × 10^−3^) has slightly more diversity than the NA gene (1.80 × 10^−3^), which is smaller than internal genes.

### 3.2. PB2 c05 and M c08 Were the Predominant Clusters in Wave 5

**Figure 2:**
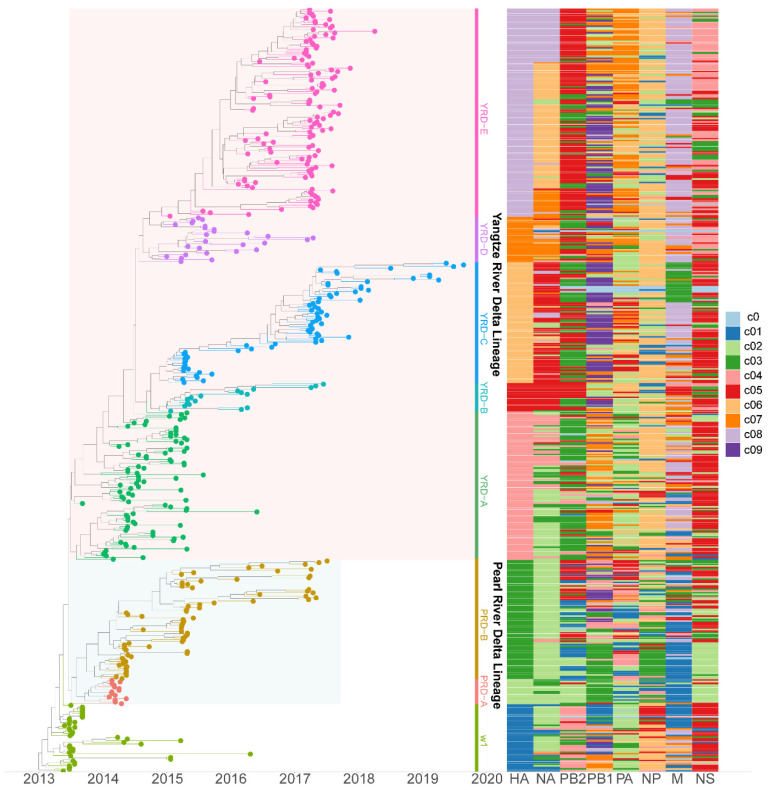
The genotypes heatmap of H7N9 viruses. Each segment’s cluster assignment is based on MCC trees with a median branch length distance threshold of 0.20 identified using PhyloPart. The left panel is the time-scaled HA tree with two lineages (Yangtze River Delta and Pearl River Delta), and tips are colored based on clusters. The right panel displays the heatmap of each segment cluster. Shared colors and numbers indicate sequences of the same segment assigned to the same cluster. The same color and number in different segments are not correlative. Detailed clusters are available in the Supplementary Table S1.

**Figure 3:**
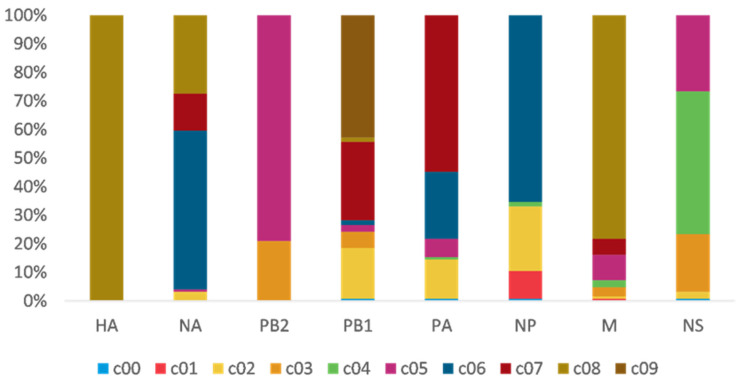
The gene clusters proportion of YRD-E. The same color and number in different segments are not correlative. PB2 c05 (79.03%) and M c08 (78.23%) are dominant clusters in the epidemic wave 5. Viruses have PB2 c05, and M c08 account for 62.10%.

To study H7N9 reassortment, we investigated the clusters of each segment for H7N9 viruses recovered from all hosts in China from 2013 to 2019 (Figure 2). Segment cluster numberings were designated, including HA (c1-c8), NA (c1-c8), PB2 (c1-c5), PB1 (c1-c9), PA (c1-c7), NP (c1-c6), M (c1-c8), and NS (c1-c5) (Figures S1–S7 and Table S1). Cluster 0 (c0) represented the phylogenetic tips that could not be assigned to a cluster. The same number in different segments is not correlative. Eight genotypes were allocated based on HA clusters: the early genotype cluster was designated as W1; the Pearl River Delta (PRD) lineage was divided into two genotypes: PRD-A and PRD-B (Figure 2); and the Yangtze River Delta (YRD) lineage designated as five genotypes: YRD-A, YRD-B, YRD-C, YRD-D, and YRD-E. LP H7N9 viruses of the YRD lineage discovered in wave 5 were mainly assigned within the YRD-E cluster, which was the dominant cluster. In the YRD-E cluster, the dominating segment clusters were c05 PB2 (79.03%) and c08 M (78.23%) (Figure 3). Viruses simultaneously containing PB2 c05 and M c08 were determined in 62.10% (77/124 in the cd99 dataset) of the YRD-E cluster. The circulating HP H7N9 AIVs were assigned to the YRD-C cluster. In the HP H7N9 virus found in waves 6 and 7, the cluster of M c03 was replaced by the c08. Reassortment has a more significant impact on the diversity of viral genotypes.

### 3.3. Intensive Intersubtype Reassortments between H7N9 and H9N2

**Figure 4:**
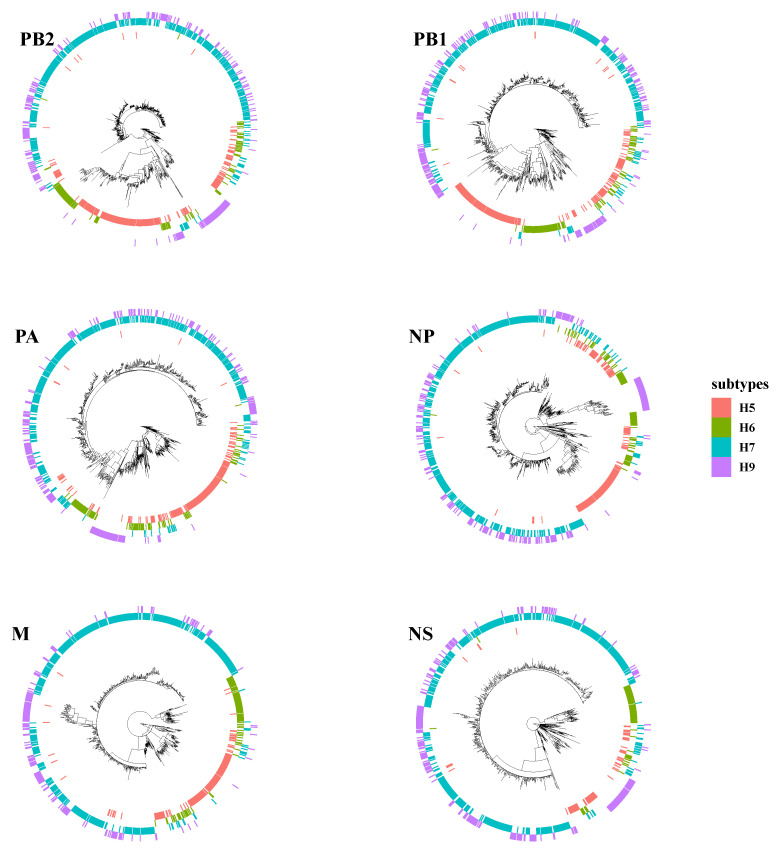
Intersubtype reassortment between H7N9 and H5, H6, and H9 subtypes of avian influenza viruses.

Phylogenetic tree analyses evaluated intersubtype reassortments between cocirculating subtypes of H5, H6, and H9. Surprisingly, the topology of H7N9 and H9N2 was clustered together in many branches on each tree of internal segments (Figure 4). H7N9 viruses were reassorted in rare cases with other subtypes of AIVs instead of H9N2. Duck-origin viruses in particular have a more complicated genetic background, with internal segments derived primarily from wild bird viruses, such as H5N6, H6N1, H6N2, H6N6, H7N2, H7N3, and H7N7. For example, the M gene of A/chicken/Guangdong/Q1/2016, the early-raised HP H7N9 obtained on 20 June 2016, is closely clustered with the M gene of the H5N6 subtype (A/duck/Yunnan/07.15 DQNPH129/2015, EPI668788). However, all these intersubtype reassortment events were predominantly within the H9N2 subtype (G57 or genotype S).

### 3.4. Dynamic and Intricate Intrasubtype Reassortment

**Figure 5:**
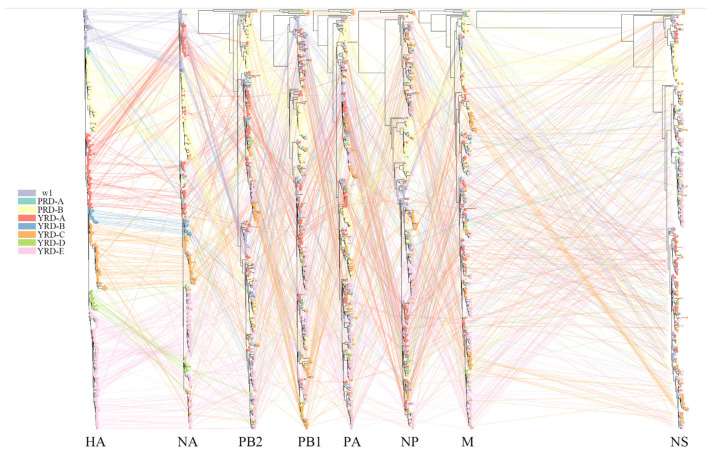
Phylogenetic incongruence analysis. MCC trees for the HA segment and all internal genes NA, PB2, PB1, PA, NP, and M from equivalent strains connect across the trees. Tips and connecting lines are colored according to the HA clusters.

**Figure 6:**
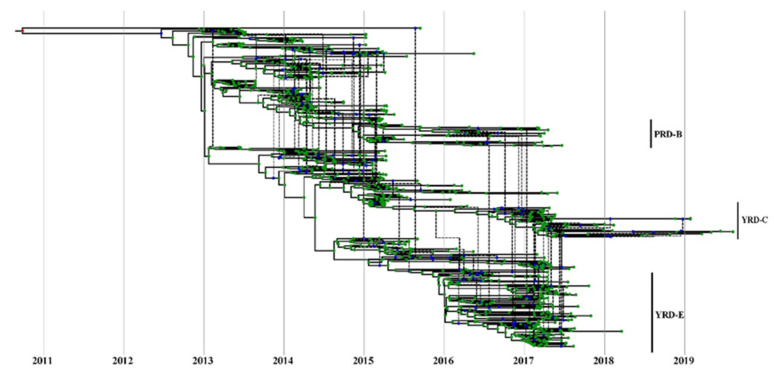
Estimate of MCC reassortment network between HA and NA genes of H7N9 viruses. Icytree visualizes the network as a base tree connected by dotted branches, indicating a reassortment event. The green dots indicate the tips of the tree.

Baltic was used to visualize inconsistencies between the MCC phylogenetic trees (tanglegram). An abundance of intralineage reassortment events was observed from the tanglegram among the 7 waves (Figure 5). Internal segments in the YRD-E (LP in wave 5) cluster were highly diverse during the wave 5 outbreak. They usually came from 2–3 main clusters on each MCC tree topology (Figure S8). Notably, part of HP H7N9 viruses in the YRD-C inherited their PB2, PA, and M segments from the YRD-E cluster (Figure S9), suggesting the LP H7N9 contributed to the genesis of HP H7N9 and frequent intralineage reassortments. There were also intralineage reassortments between the YRD and PRD lineages. For instance, some YRD-C HP H7N9 viruses obtained the NA gene from the cocirculating PRD-B cluster. Intralineage reassortment events between the YRD and PRD lineages were also commonly and consistently identified throughout all H7N9 epidemic waves by BEAST2/CoalRe (Figure 6). Especially, many intralineage reassortment instances were also determined in waves 2–3 and 5. Although the H7N9 population was severely decreased at the time, the HP H7N9 virus isolated in waves 6 and 7 continued to reassort. For instance, the M gene of A/chicken/Shanxi/SX0256/2019 came from c07 rather than the c03. The M c03 has been the dominant cluster in the previous prevalent viruses since 2018 (Figure 2). Of note, none of the genotypes was predominant in any of the seven epidemic waves. Our results demonstrated that China’s H7N9 virus has a dynamic and intricate intrasubtype reassortment pattern.

### 3.5. HA and NA Have the Lowest Reassortment Rate

**Figure 7:**
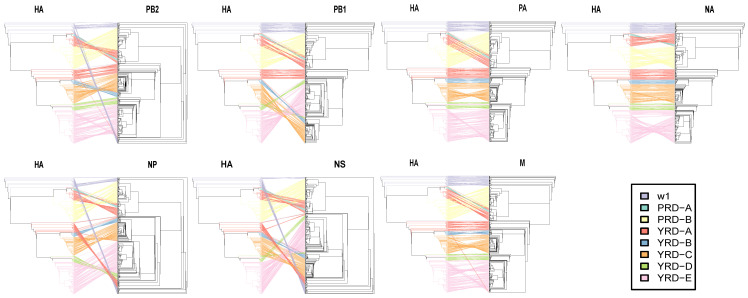
Evolutionary relationships of each gene segment with HA. Incongruence phylogenetic analysis shows interclade reassortment between the HA segment and seven additional genes (NA, PB2, PB1, PA, NP, and M). Equivalent strains connect across the trees. Tips and connecting lines are colored according to the HA clusters. Unentanglement is used to minimize crossings of the hybridization network between the paired trees. The values are determined by the degree of inter-segment reassortment.

To measure the severity of reassortment over the entire genome, we untangled the tanglegram between HA and the paired trees shown as untanglement values (the bigger, the worse. Figure 7). The unentanglement values between HA and paired segments (NA, PB2, PB1, PA, NP, M, and NS) were 0.0381, 0.1997, 0.2340, 0.0591, 0.3234, 0.0672, and 0.4894, respectively. The lowest untanglement value (0.0381) was found between HA and NA, whereas the highest value was found between HA and NS (0.4894).

### 3.6. High Reassortment Rate in Waves 3 and 5

GiRaF and RDP analyses found that 217 of 454 strains H7N9 (cd99 dataset) were involved with reassortment events (Table S2). Following that, we computed the reassortment rates in different waves. High reassortment rates were discovered in waves 3 (0.76) and 5 (0.79). Wave 1 is notable for having the lowest reassortment rate (0.10).

### 3.7. PB2 and PA Shared Coevolutionary History

**Figure 8:**
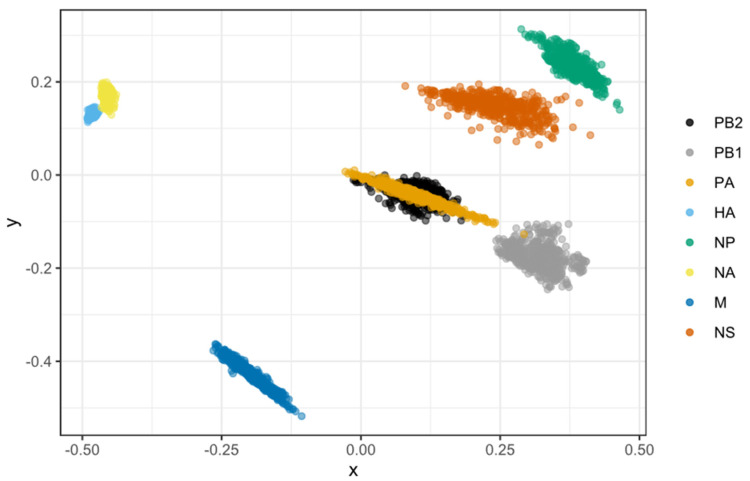
Correlations in time to the most recent common ancestor (tMRCA) among H7N9 viral segments as depicted by a multidimensional scaling (MDS) graphic. MDS enables a two-dimensional representation of the total level of cross-correlation between all segments. Cloud points represent phylogenetic uncertainty based on 500 trees for each segment sampled in the program of BEAST, with pairwise comparisons to other segments limited to viruses sampled in the same year. In the absence of reassortment, segments are likely to have highly correlated TMRCAs due to shared evolutionary history showing as overlapping dots. Reversely, segments split up by reassortment are predicted to inhabit various plot regions. Only the first two scaling dimensions are visible.

MDS allows for the two-dimensional depiction of the total degree of cross-correlation between all segments, with overlap between observations indicating shared evolutionary history (i.e., linkage) between segments. In comparison, segments that split up due to reassortment are expected to occupy separate plot areas. We found that, except for the PB2 and PA segments, the rest of the H7N9 segments were very distinct (Figure 8). The segments PB1, M, NP, and NS did not show any association, indicating that they did not have any coevolutionary relationship. The PB2 and PA genes almost completely overlapped, indicating they shared a strong evolutionary history (i.e., linkage). In comparison, the capsid proteins of HA and NA only had a weak association.

### 3.8. High Selection Pressure Acting on HA, NA, NS1, and M2

The natural selection acting on all coding regions was estimated using the dN/dS ratio. Four site-level detection methods (FEL, SLAC, FUBAR, and MEME) were used to assess positive and negative selection codons. M2 (0.5398), NS1 (0.3087), HA (0.2656), and NA (0.2946) underwent more substantial selection pressure than other segments, whereas M1 protein (0.0752) was the lowest, according to the overall dN/dS values estimated using SLAC (Table 1). Except for M2, NS1, and NEP, the other internal proteins were unaffected by higher purifying selectivity. MEME was used to find more positive selection sites by identifying fixed and sporadic positively chosen codons. As a result, HA (14 codons) and NA (13 codons) had more sites under positive selection pressure than other coding regions. PB2 (10 codons), NS1(10 codons), PB1 (7 codons), and PA (6 codons) also detected many positive selection sites.

## 4. Discussion

We estimated the inter- and intra- reassortment, reassortment patterns, and adaptative evolution of H7N9 viruses from waves 1–7. Our study found that H7N9 viruses have undergone considerable changes by reassortment, which had a significant impact on the H7N9 genomic composition since its emergence. Notably, the internal genes presented more diversified features than surface genes, and even the surface genes had higher substitution rates. Numerous reassortment events were determined in our study, resulting in a high level of genetic diversity, especially among the internal genes. The dynamic and intricate reassortments may shape the epidemiology and genomic evolution of the H7N9 virus and contribute to its genetic diversity.

The H7N9 haemagglutinin gene evolved faster (6.51 × 10^−3^ substitutions/site/year) than the seasonal influenza virus, with an average rate of 3.41 × 10^−3^ [59]. This substitution rate is similar to the H5N1 under vaccination pressure (6.13 × 10^−3^–8.87 × 10^−3^) but faster than H5N1 without vaccination pressure [60]. According to recent studies, the nucleotide substitution rate of the H7N9 HA gene under vaccination pressure increased to 1.963 × 10^−2^ during 2018–2019 [61]. Considering that reassortment usually leads to a transient increase in the substitution rate [62], the frequency of reassortments may also contribute to the rapid evolution of H7N9. According to other research, external genes of the influenza virus usually have evolved at faster evolutionary rates than its internal genes [63,64], resulting in more genetic diversity in surface genes. However, the external genes of the H7N9 virus showed less genetic diversity than its internal segments. This contradiction implies that H7N9 internal genes were reassorted frequently rather than in a single event since its emergence.

In the study of intersubtype reassortments, we found that the H7N9 virus was primarily associated with the cocirculating H9N2 virus (genotype S or G57). In contrast, they were rarely reassorted with other AIV subtypes. The H9N2 and H7N9 viruses have been cocirculating in China since 2013. Therefore, numerous opportunities existed for their coinfections. Several investigations in the first five epidemic waves also confirmed the high prevalence of H7N9/H9N2 coinfection in chickens [15,25,27,65]. Influenza virus reassortment occurs with high frequency without segment mismatch [66]. However, most reassortment viruses produced from divergent parental strains are also frequently outcompeted by one or both parental viruses [2]. Specific reassortment genotypes may be inefficient in forming due to incompatibility between the heterologous RNA packaging signals [2,67]. Considering the high genetic similarity between the internal segments of H7N9 and H9N2 AIVs, it may be the biological basis for the high frequency of intersubtype reassortment in the absence of mismatches between their packaging signals. Heterogeneous genome packaging signals combinations other than H7N9/H9N2 viruses may be deleterious or decrease fitness under natural conditions [68], forcing the reassorted progeny virus particles to be eliminated through purifying selection. Therefore, the high frequency of intersubtype reassortants between H7N9 and H9N2 AIVs could be expected.

The LP H7N9 virus, unlike the other LP AIV, may cause serious illness in humans and other mammalian species, demonstrating its exceptional fitness in mammalian hosts [69]. The fifth wave of 2016–2017 was the biggest epidemic to date, with nearly the same number of human cases (*n* = 758) as the sum of the previous four outbreaks [36]. Nonetheless, most human infections in wave 5 were caused by the LP H7N9 virus, which is primarily associated with YRD-E. Our investigation found that gene clusters PB2 c05 and M c08 have a large proportion of YRD-E cluster strains, which mainly circulated in wave 5 despite a highly diverse genetic background. Our previous studies showed that the reassortments with PB2 and M genes from genotype S H9N2 caused attenuated progeny of H5Nx and H7N9, resulting in optimizing viral fitness viruses in mice [70] and chicken [71]. Therefore, we assume that PB2 c05 and M c08 might optimize the viral fitness and contribute to the wave 5 outbreak. Aside from the PB2 and M, NP c06 and PA c07 have a certain ratio in YRD-E. It is also impossible to overlook their contribution to viral fitness.

In terms of intralineage reassortment, we found that the intralineage reassortment occurs considerably frequently rather than as a single event. Usually, intralineage reassortment is subjected to severe negative selection, which becomes more pronounced as the genetic distance between donor strains increases [72]. Due to the remarkable similarity in their internal genes and genomic packaging signals between the H9N2 and H7N9 viruses, the high compatibility between their internal genes makes the reassorts rise rather naturally. According to our findings, the YRD-E lineage contributed its PB2, PA, and M gene to the YRD-C lineage, dominated by the HP H7N9 viruses. The frequent reassortment leads to such a diversity of genotypes of H7N9. Similarly, Cui et al. documented 27 H7N9 genotypes within 3 months of H7N9 emergence [35]. Nonetheless, it should be highlighted that intralineage reassortment between pretty similar internal gene cassettes is likely to be underestimated. Using tanglegram to estimate the reassortment in phylogenies from high similarity sequences might underestimate or exaggerate reassortment events [46]. Combined with additional pieces of evidence [27,28,29], we confirmed that the intricate intralineage genetic reassortment of the H7N9 virus had occurred frequently since its introduction.

Usually, wild birds are the primary source of AIV reassortant [73]. However, we found that H7N9 reassortment occurred mainly in the chicken host rather than in wild birds. The most likely reason was that H7N9 and H9N2 were primarily cocirculated in poultry, while H7N9 only had sporadic spillover to wild birds [74]. The other reason is that national surveillance relied predominantly on passive reporting systems and less active and systemic surveillance in wild birds [75]. Therefore, the “sampling strategy” in the H7N9 surveillance may affect the reassortment interpretation.

Many intersubtype reassortments took place between HA and NA in waves 2–3 and 5, coinciding with the periods of extensive intrasubtype and intralineage reassortment. Theoretically, H7N2 and H9N9 should have a high probability of detection since the extensive interlineage reassortment between H7N9 and H9N2. However, the detection of the H7N2 and H9N9 virus subtypes was limited. Even the reassorted H9N9 virus in the laboratory has shown increased fitness features in poultry [76]. Although intersubtype reassortments between H7 and N9 were frequently detected in waves 2–3 and 5, the frequency of reassortment between HA and NA remained low compared to HA with other segments, demonstrating a robust functional balance between H7 and N9. Similar to other avian influenza viruses in poultry and wild bird populations, the NS gene encoding the non-structural protein has a more divergent phylogeny and high reassortment rates [73,77].

Reassortment in the influenza virus is not a random process, which is consistent with observations from other segmented viruses [4,67]. Except for the PB2 and PA segments, the other H7N9 segments did not share their evolutionary history segment. However, the HA-NA capsid proteins and the PB1-PB2/PA RNA polymerase complex proteins only had a weak association. The times to most recent common ancestor (tMRCA) were consistent in PB2 and PA, indicating they were less amenable to reassortment than the other segments in history. This inherited linkage also implies physical or biochemical interactions between their encoded proteins since epistatic interactions result in tighter evolutionary connections. According to a recent study, reintroducing PB2 and PA with adaptive mutations from cocirculating H9N2 in 2015 resulted in a novel H7N9 genotype, which increased polymerase activity and became dominant in the fifth H7N9 virus epidemic [36]. Because the internal segments primarily arose from genotype S H9N2 [34] (similarly with G57 genotype H9N2 [36]), this linkage PB2-PA pattern most likely originated from H9N2. A previous study found that a coadapted PB1-PB2-HA gene pattern was established during influenza B interlineage reassortment, which has been demonstrated to be critical for whole-genome fitness [78]. As a result, we assume that the particular PB2-PA pattern might confer a specific advantage to the H7N9/H9N2-like gene cassette. Further studies are warranted.

In addition to M2, NS1, and NEP, high dN/dS values were found in the HA and NA proteins (0.5398, 0.3087, 0.2766, 0.2656, and 0.2946, respectively), which were generally in agreement with the previous study in H3N2 [46]. Surface proteins (HA, NA, and M2) are typically more sensitive to positive selection and evolve more rapidly than internal genes, especially when the influenza virus circulates in a naive population [63,64]. The reassortment events lead to a transient increase in the rate of amino acid replacements on the descendant phylogenetic branches [79]. Therefore, the extensive reassortment might increase the H7N9 evolutionary rate and result in rapid adaptive evolution at the molecular level.

In summary, our phylogenetic analyses revealed the comprehensive genetic evolution of the H7N9 viruses. The particular reassortment patterns indicate that the H9N2-original internal gene constellation has superior compatibility to the genesis and evolution of H7N9 over the other AIV subtypes. The H7N9 viruses were further diversified by frequent inter- and intra-lineage reassortment events with adaptative mutation, leading to successful H7N9 genotypes. The evolution and epidemiology of the H7N9 virus in China may be shaped by reassortment and adaptive mutations. Further research is needed to understand the dynamics of reassortment of the circulating H7N9 virus.

## Figures and Tables

**Table 1:** Selection pressures and positively and negatively selected codons of coding regions of H7N9 viruses circulating between 2013 and 2019.

Coding Region	No. of Codons	dN/dS	No. of Selected Sites (% of Codons)				
			Positively Selected				Negatively Selected (SLAC)
			SLAC ^a^	FUBAR ^b^	FEL ^c^	MEME ^d^	
PB2	759	0.1200	4	4	4	10	670 (88.16%)
PB1	757	0.0977	5	6	6	7	657 (75.60%)
PA	716	0.1137	7	4	8	6	573 (79.92%)
HA [38]	564	0.2656	16	13	16	14	374 (66.19%)
NP	498	0.1026	2	1	4	3	413 (82.77%)
NA [38]	465	0.2946	13	8	15	13	280 (60.09%)
M1	252	0.0752	0	1	1	1	190 (75.10%)
M2	97	0.5398	6	7	8	3	26 (26.53%)
NS1	217	0.3087	13	6	14	10	115 (52.75%)
NEP	121	0.2766	0	1	1	2	55 (45.08%)

^a^ *p*-value < 0.05; ^b^ posterior probability of ≥0.9; ^c^ *p*-value < 0.1; ^d^ *p*-value < 0.05

## Data Availability

Data are derived from the public database and 10.1016/j.meegid.2021.104993.

## Supplementary Materials

The following supporting information can be downloaded at: https://www.mdpi.com/article/10.3390/v14061256/s1. Table S1, PhyloPart clusters; Figures S1 to S7, H7N9 segments phylopart cluster tree; Table S2, Girad and RDP; Figure S8, Phylogenetic incongruence analysis of YRD-E; Figure S9, Phylogenetic incongruence analysis of YRD-C.
