# Supplementary figures and images for: Genome-Wide Reassortment Analysis of Influenza A H7N9 Viruses Circulating in China during 2013–2019
Source: Viruses. 2022 Jun 9;14(6):1256. doi: 10.3390/v14061256 (PMC9230085; doi:10.3390/v14061256)

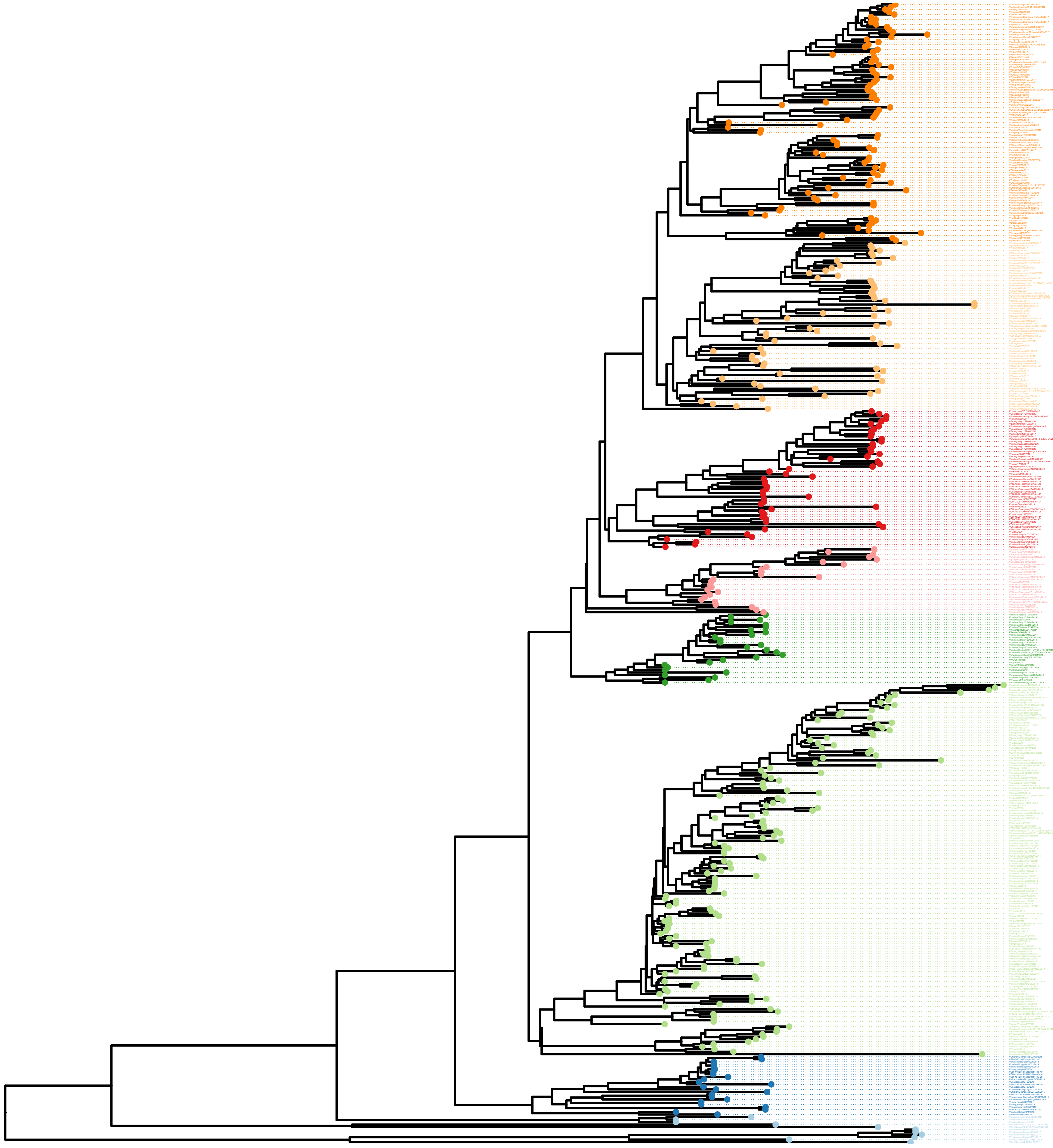

- clades
- c00
  - c01
  - c02
  - c03
  - c04
  - c05
  - c06
  - c07

Supplement: Supplementary file 1 [file viruses-14-01256-s001.zip › Figure_S4_H7N9_PA_cd99_phylopart_cluster.pdf]

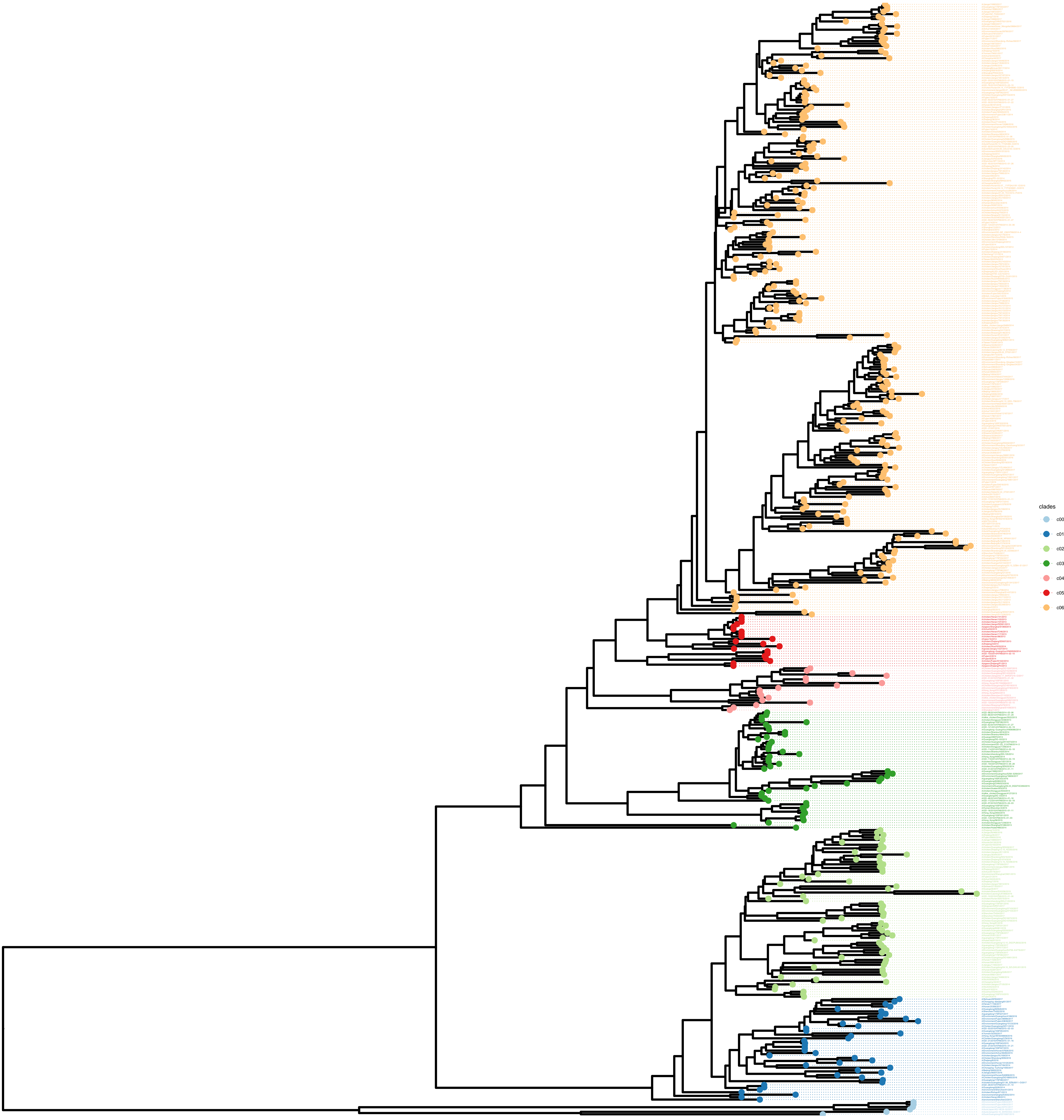

Supplement: Supplementary file 1 [file viruses-14-01256-s001.zip › Figure_S5_H7N9_NP_cd99_phylopart_cluster.pdf]

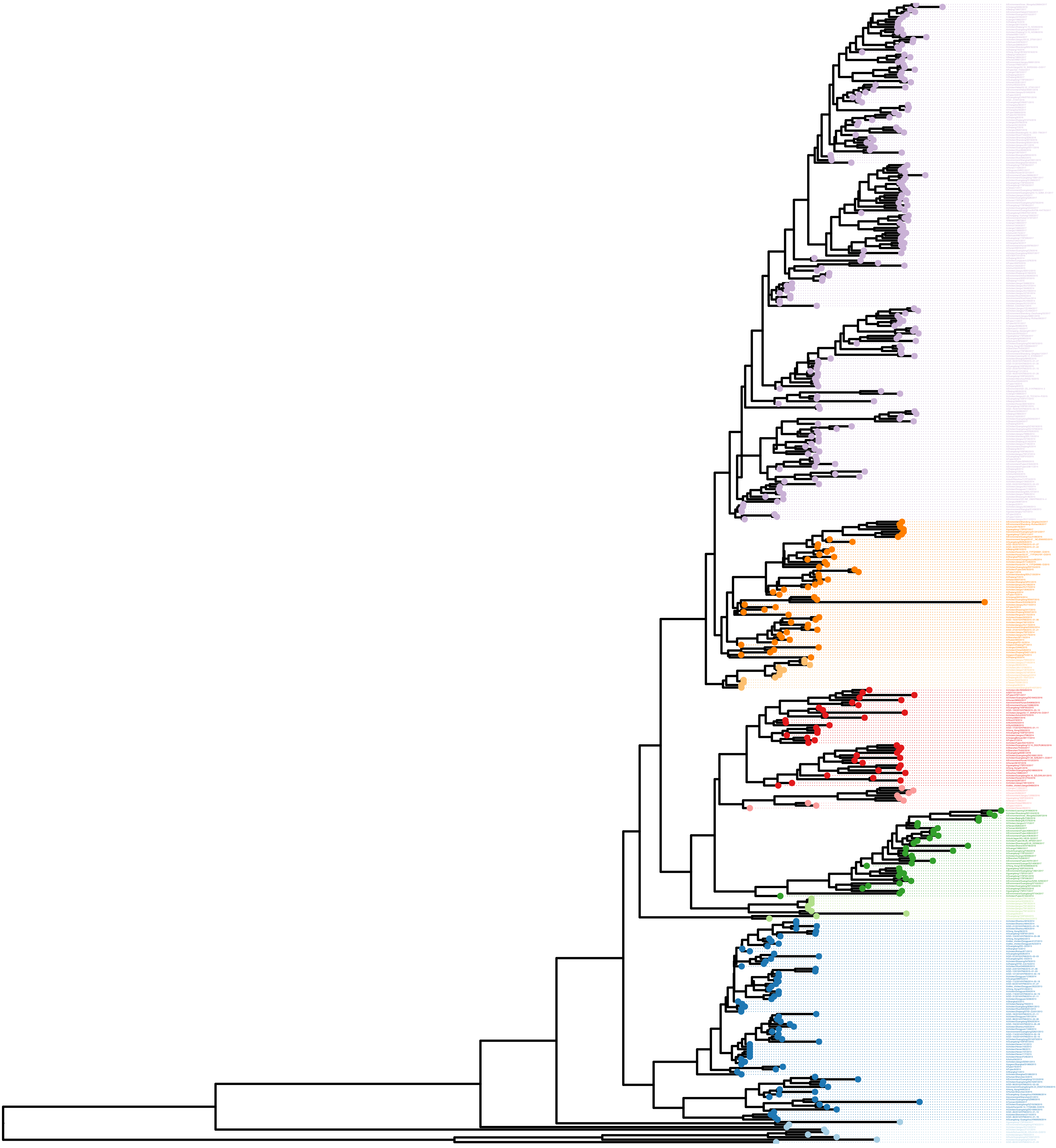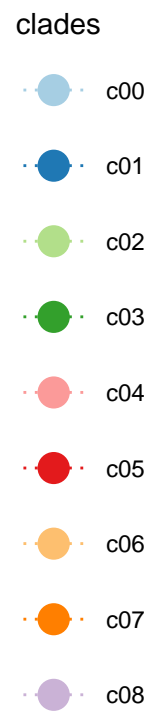

Supplement: Supplementary file 1 [file viruses-14-01256-s001.zip › Figure_S6_H7N9_M_cd99_phylopart_cluster.pdf]

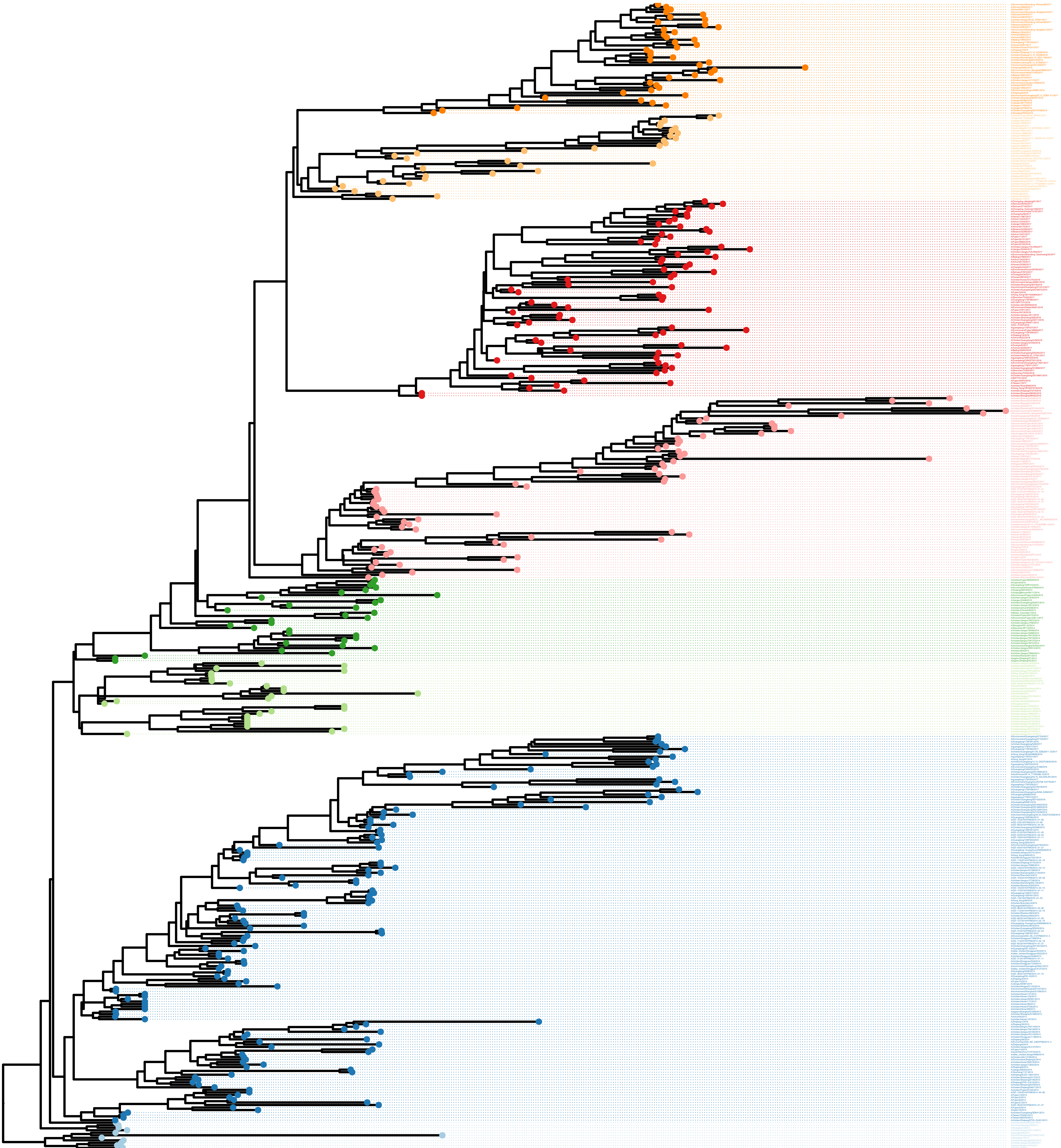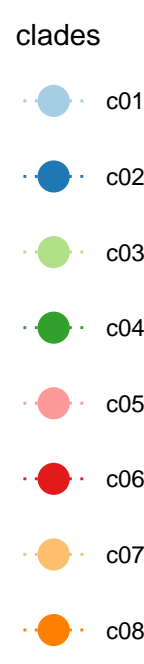

Supplement: Supplementary file 1 [file viruses-14-01256-s001.zip › Figure_S1_H7N9_NA_cd99_phylopart_cluster.pdf]

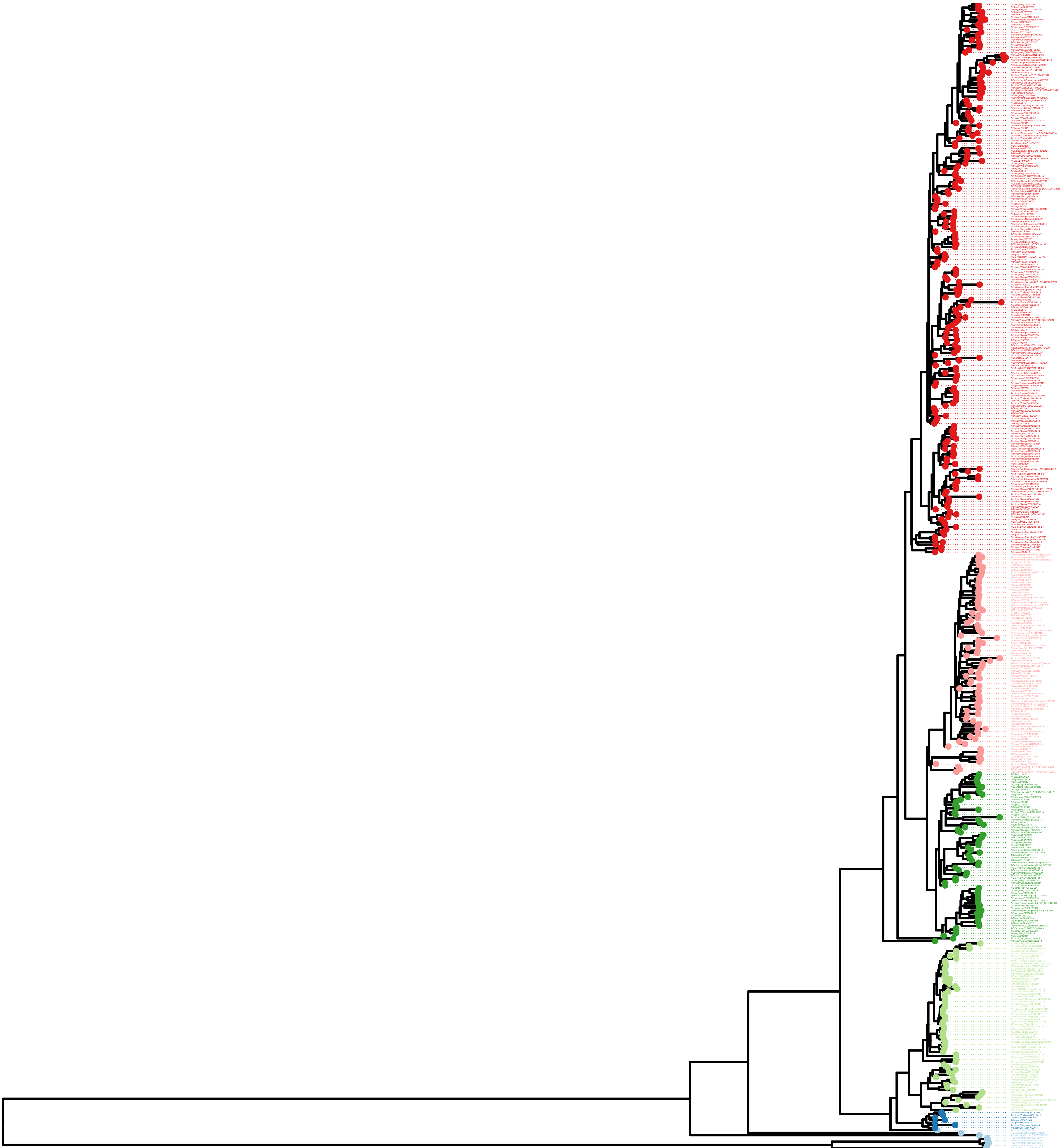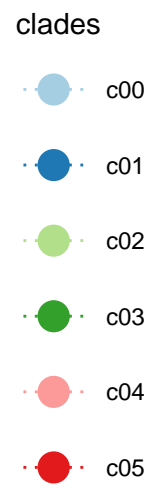

Supplement: Supplementary file 1 [file viruses-14-01256-s001.zip › Figure_S7_H7N9_NS_cd99_phylopart_cluster.pdf]

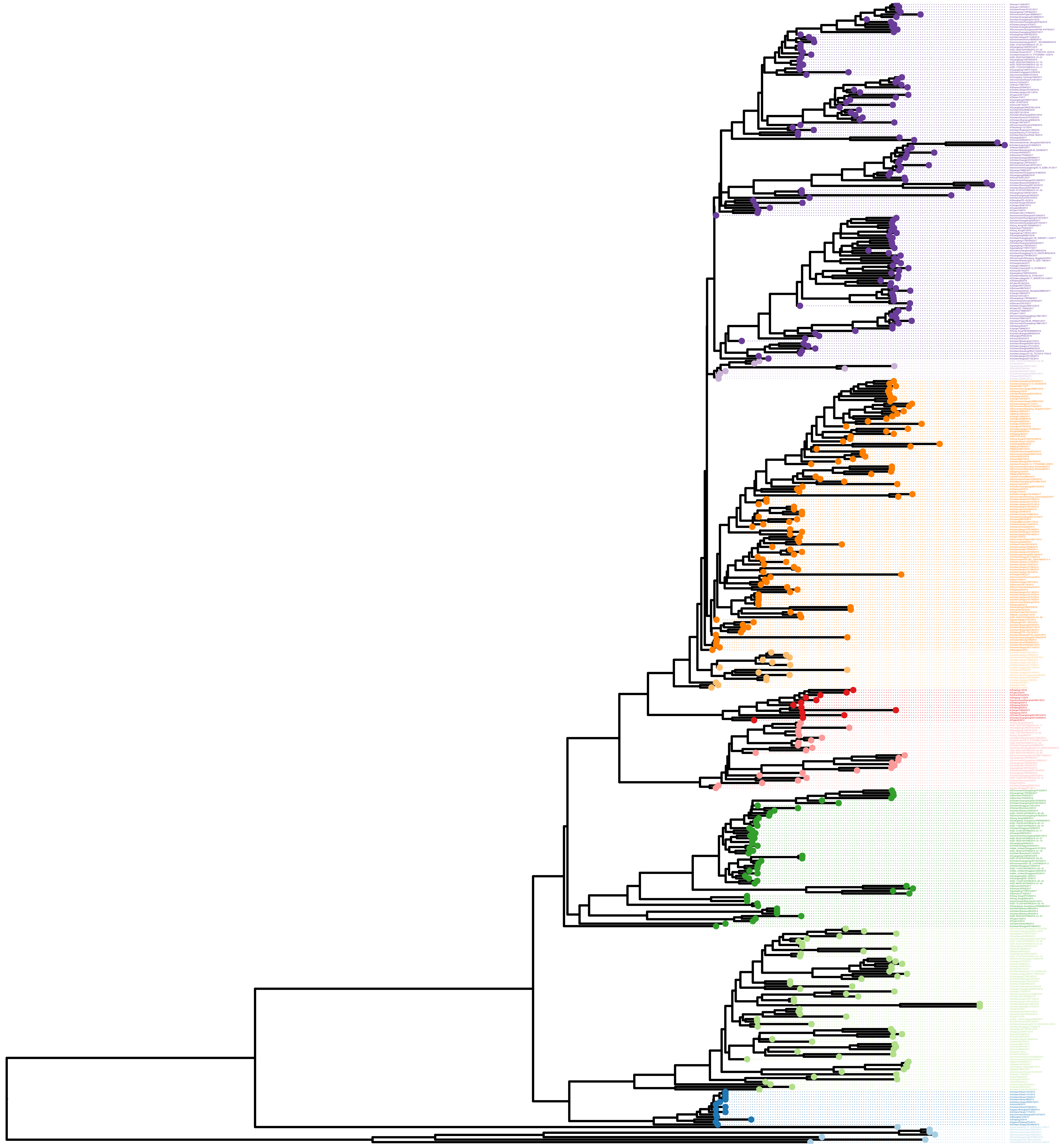

Supplement: Supplementary file 1 [file viruses-14-01256-s001.zip › Figure_S3_H7N9_PB1_cd99_phylopart_cluster.pdf]

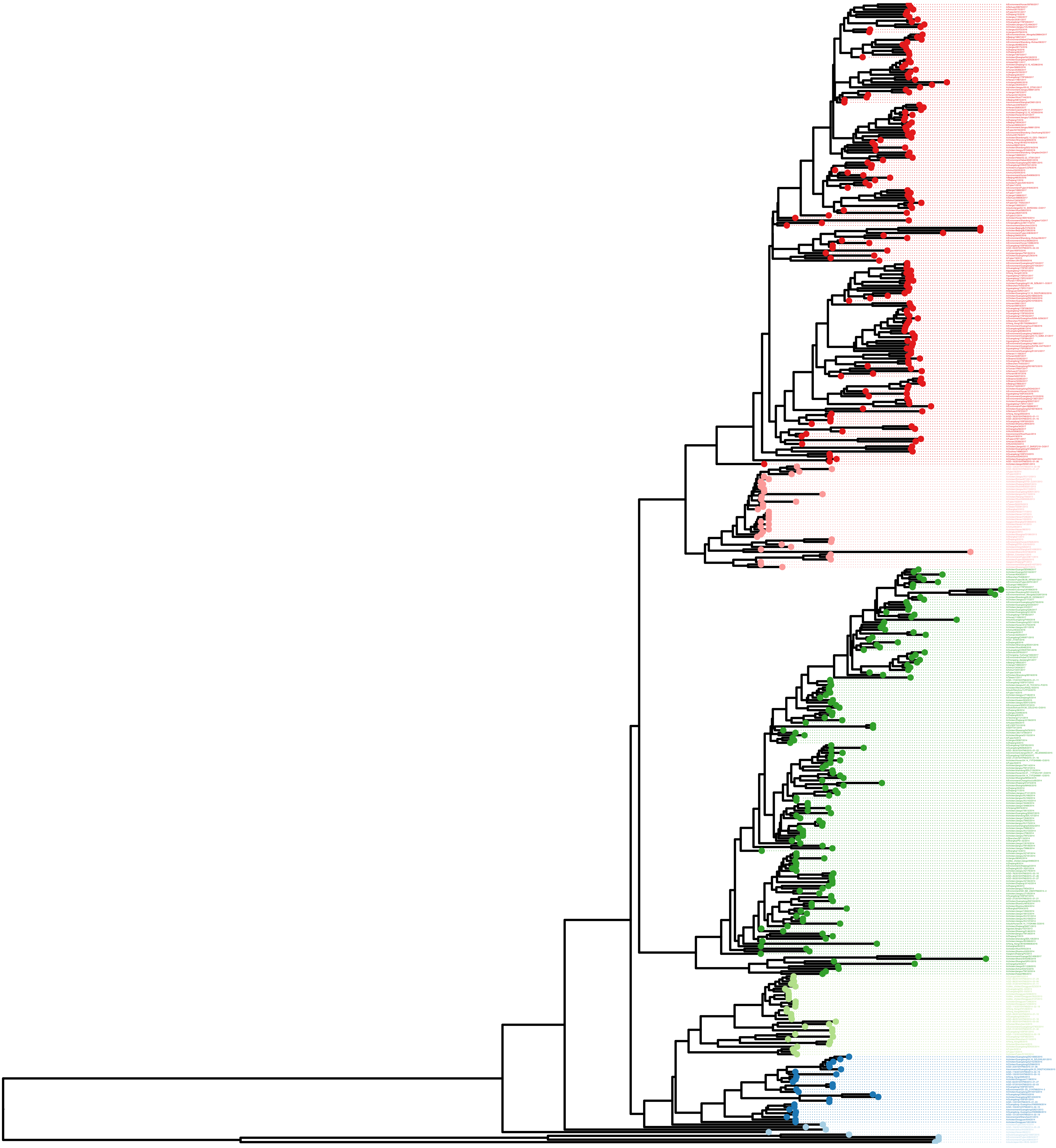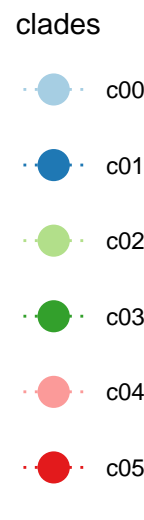

Supplement: Supplementary file 1 [file viruses-14-01256-s001.zip › Figure_S2_H7N9_PB2_cd99_phylopart_cluster.pdf]
