# Supplementary material for: Genome-Wide Reassortment Analysis of Influenza A H7N9 Viruses Circulating in China during 2013–2019
Source: Viruses. 2022 Jun 9;14(6):1256. doi: 10.3390/v14061256 (PMC9230085; doi:10.3390/v14061256)
Supplement: Supplementary file 1 [file Figure_S9_baltic_mcc_YRD-C.pdf › Figure_S9_baltic_mcc_YRD-C.pdf]

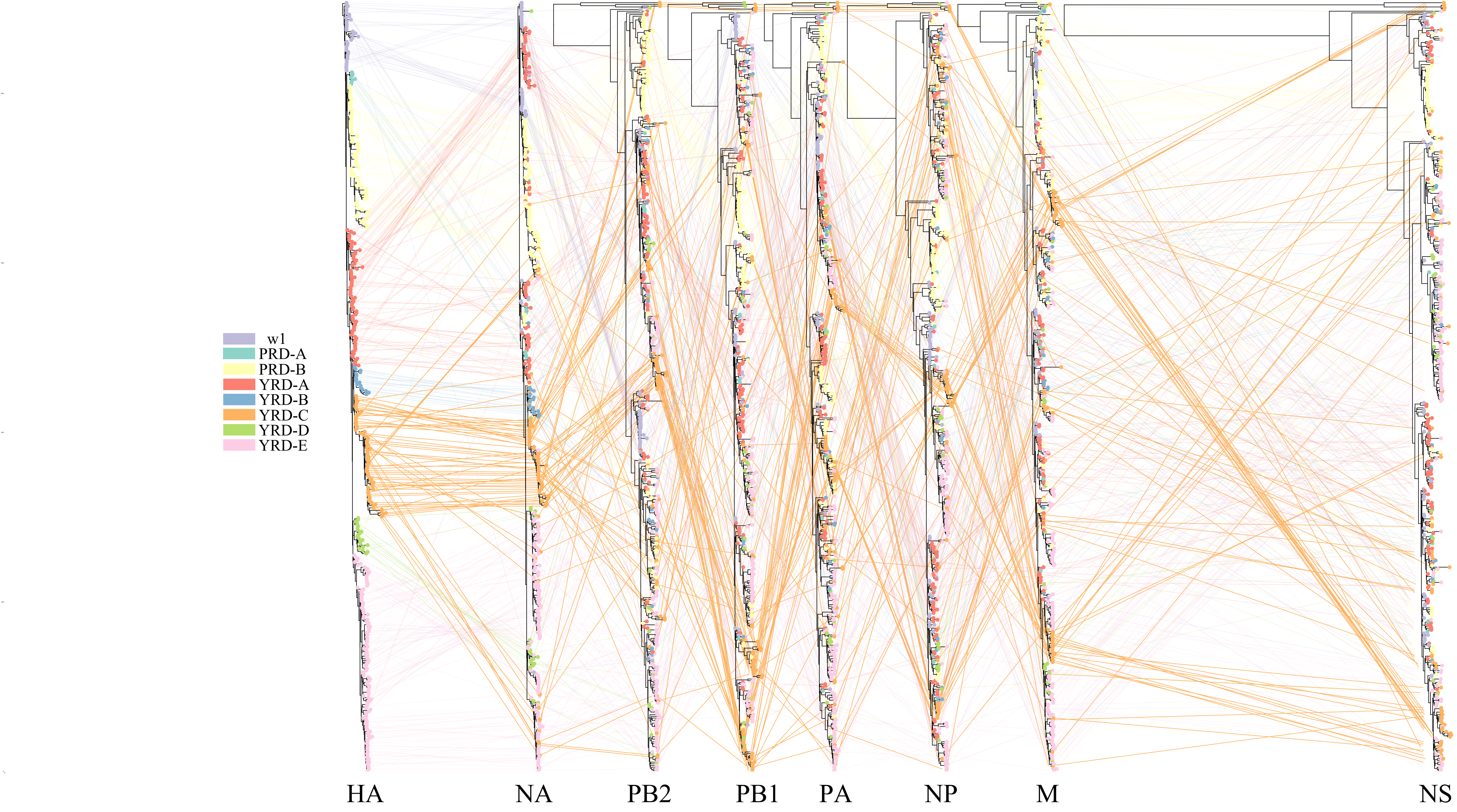

w1
PRD-A
PRD-B
YRD-A
YRD-B
YRD-C
YRD-D
YRD-E
HA
NA
PB2
PB1
PA
NP
M
NS
